# Cardioneuroablation for vasovagal syncope: insights on patients’ selection, centre settings, procedural workflow and endpoints—results from an European Heart Rhythm Association survey

**DOI:** 10.1093/europace/euae106

**Published:** 2024-05-23

**Authors:** Diego Penela, Antonio Berruezo, Laurent Roten, Piotr Futyma, Sergio Richter, Giulio Falasconi, Rui Providencia, Julian Chun

**Affiliations:** Humanitas Research Center, Milano, Italy; Heart Institute, Teknon Medical Center Barcelona, Spain; Heart Institute, Teknon Medical Center Barcelona, Spain; Department of Cardiology, Inselspital-Bern University Hospital, University of Bern, Bern, Switzerland; St. Joseph’s Heart Rhythm Center Rzeszow and Medical College, University of Rzeszow, Rzeszow, Poland; Heart Center Dresden, University Hospital, Technical University Dresden, Dresden, Germany; Humanitas Research Center, Milano, Italy; Heart Institute, Teknon Medical Center Barcelona, Spain; St Bartholomew’s Hospital, Barts Heart Centre, Barts Health NHS Trust, London, UK; Cardioangiologisches Centrum Bethanien, Agaplesion Bethanien Krankenhaus, Frankfurt, Germany

**Keywords:** Survey, Cardioneuroablation, Vasovagal Syncope

## Abstract

**Aims:**

Cardioneuroablation (CNA) is a catheter-based intervention for recurrent vasovagal syncope (VVS) that consists in the modulation of the parasympathetic cardiac autonomic nervous system. This survey aims to provide a comprehensive overview of current CNA utilization in Europe.

**Methods and results:**

A total of 202 participants from 40 different countries replied to the survey. Half of the respondents have performed a CNA during the last 12 months, reflecting that it is considered a treatment option of a subset of patients. Seventy-one per cent of respondents adopt an approach targeting ganglionated plexuses (GPs) systematically in both the right atrium (RA) and left atrium (LA). The second most common strategy (16%) involves LA GP ablation only after no response following RA ablation. The procedural endpoint is frequently an increase in heart rate. Ganglionated plexus localization predominantly relies on an anatomical approach (90%) and electrogram analysis (59%). Less utilized methods include pre-procedural imaging (20%), high-frequency stimulation (17%), and spectral analysis (10%). Post-CNA, anticoagulation or antiplatelet therapy is prescribed, with only 11% of the respondents discharging patients without such medication. Cardioneuroablation is perceived as effective (80% of respondents) and safe (71% estimated <1% rate of procedure-related complications). Half view CNA emerging as a first-line therapy in the near future.

**Conclusion:**

This survey offers a snapshot of the current implementation of CNA in Europe. The results show high expectations for the future of CNA, but important heterogeneity exists regarding indications, procedural workflow, and endpoints of CNA. Ongoing efforts are essential to standardize procedural protocols and peri-procedural patient management.

## Graphical Abstract

**Graphical Abstract:**
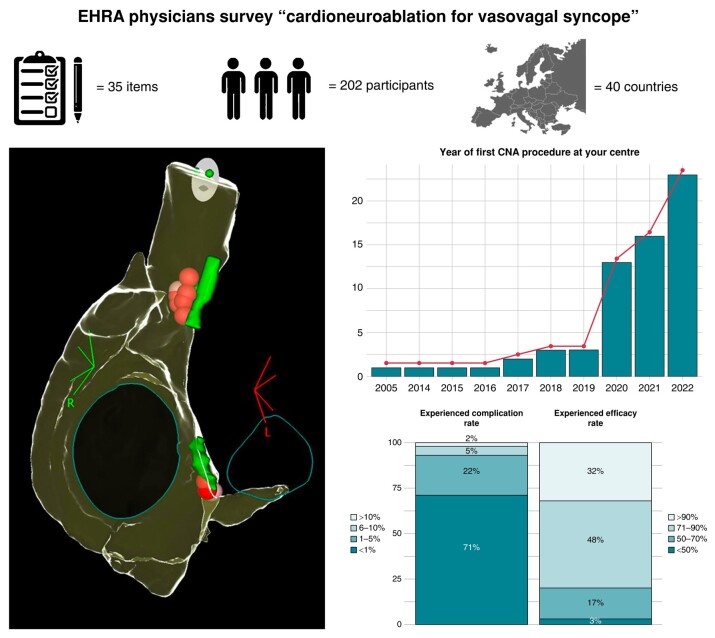

## Introduction

Vasovagal syncope (VVS) is a prevalent condition often linked to a decreased quality of life.^1–4^ Although VVS is not connected to increased mortality, a substantial proportion of patients face frequent recurrent episodes.^5,6^ Cardioneuroablation (CNA) is a recently described catheter-based intervention for VVS that consists in the modulation of the cardiac autonomic nervous system.^7^ The intrinsic part of the parasympathetic system includes the ganglionated plexuses (GPs) on the epicardial surface of the left and right atria. By means of endocardial catheter ablation (CA), these GPs can be targeted, with the aim to modify the parasympathetic efferent branch of the reflex pathway and to mitigate the pronounced cardioinhibitory response in patients with cardioinhibitory and mixed VVS.^8–10^ After the initial description of this therapy in 2005,^11^ several observational studies have reported that CNA significantly reduces VVS recurrence rates.^7,8,12–17^ The growing number of publications on this topic has generated increased interest in CNA and has also resulted in the wider adoption of this novel therapy for VVS over the past few years. Nevertheless, several gaps in knowledge persist, particularly regarding clinical indications, ablation methodology, and long-term outcomes.

The primary objective of this survey is to offer a thorough examination of the day-to-day utilization of CNA in Europe. This includes an assessment of the existing practice and the procedural techniques employed in various electrophysiology (EP) laboratories. Furthermore, the survey seeks to pinpoint any obstacle that might impede the adoption of CNA in clinical practice, while also identifying the research requirements of the EP community for advancing this field in the future.

## Methods

This survey was an online questionnaire created by the European Heart Rhythm Association (EHRA) Scientific Initiative Committee and sent out by the EHRA. It was distributed via social media and national cardiac and EP societies and their members. The survey was accessible for 6 weeks (starting from July 2023) and its participation was physician based, anonymous, and voluntary.

The survey was split into two sections, encompassing a total of 35 questions. The initial section, counting 14 questions, focused on the VVS management, including routine clinical practice of syncope treatment, available facilities, availability of CNA, the number of CNA cases performed, and the expectations for this therapy in the future.

The second part of the survey included up to 21 questions and was specifically designed for centres actively conducting CNA procedures. It centred on various aspects such as candidate selection, procedural setting and workflow, and the outcomes of CNA.

The survey (available as Supplementary material) was elaborated by the principal investigator of the study and revised and approved by members of the EHRA Scientific Initiatives Committee.

## Results

### General aspects

After a period of 6 weeks, a total of 202 participants from 40 different countries replied to the survey. Supplementary material online, *Figure S1* shows the response by countries. The majority of participants (38%, 70/183) indicated to perform 150–500 CA procedures in general at their centres per year. One-fourth (51/183) indicated to perform between 501 and 1000 CA/year. Only 28 responders perform more than 1000 CA/year. Regarding pacing procedures, 54% (98/183) of participants reported to implant 150–500 device/year, while almost one-third (65/183) indicated to implant more than 500 devices/year. Among responders, 45% (82/181) are actually working in a university hospital, whereas 27% (49/181) work in a private setting. Only 33% (66/202) of responders claimed not to have heart surgery available at their primary workplace.

### Syncope management

**Figure 1:**
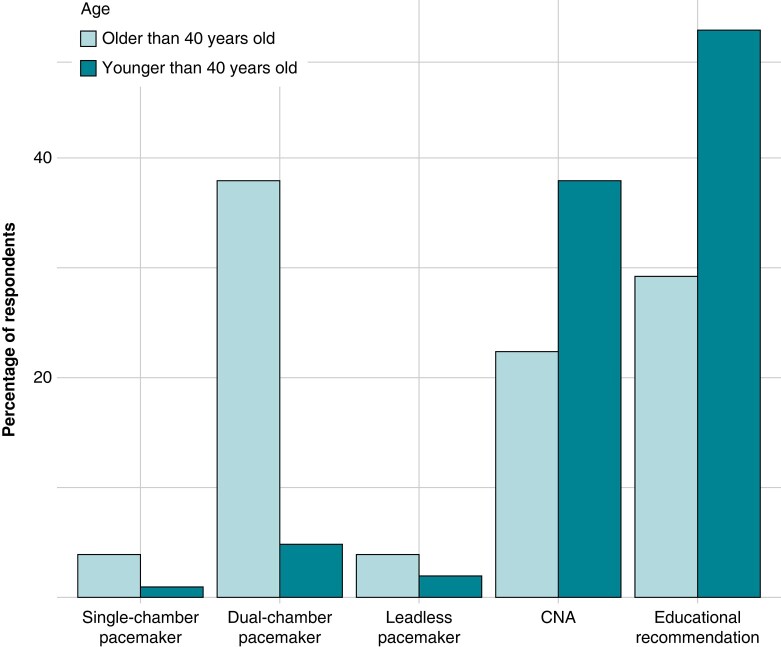
First-line therapeutic approach to VVS according to the patient age. The grouped bar chart displays the percentage of respondents who treated their patients with single single-chamber pacemaker, dual-chamber pacemaker, leadless pacemaker, CNA, and educational recommendation. CNA, cardioneuroablation.

Only one-third (29%, 59/202) of participants reported to have a dedicated syncope unit in their institution, and 54% (109/202) of respondents affirmed to perform tilt table test as part of the routine evaluation of syncope. Participants were asked for their first-line approach in patients with recurrent cardioinhibitory VVS. *Figure 1* illustrates that sequential atrioventricular DDD pacing was the preferred therapeutic option in patients aged over 40 years, while educational recommendations were favoured in patients younger than 40.

The survey participants were questioned regarding the clinical efficacy of CNA in the context of VVS. A majority (65%, 116/178) expressed the view that CNA is beneficial in cases of VVS with a cardioinhibitory response during tilt tests in young patients. However, this percentage decreased to one-third (56/178) of respondents when this clinical scenario was expanded to include patients of all age groups. A notable percentage of respondents (58%, 104/178) believe that CNA remains valuable in the management of patients experiencing recurrent VVS with documented vagally induced high-degree atrioventricular block. However, in cases of cardioinhibitory carotid sinus syndrome, the perceived utility of CNA dropped to 38% (68/178). Lastly, only a small proportion (5%, 9/178) of the participants do not see a role for CNA in VVS management.

**Figure 2:**
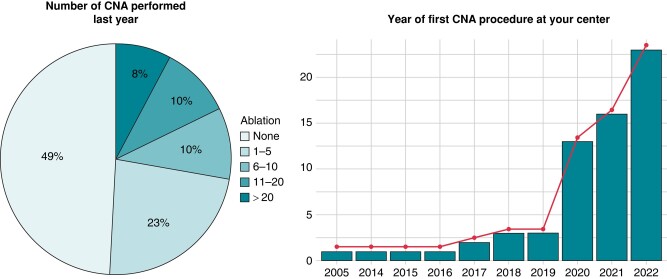
Number of CNA performed annually and year of the first CNA procedure. The pie chart displays the percentage of respondents according to number of CNA performed last year; the bar chart reported the number of centres according to the year of the first CNA performed. CNA, cardioneuroablation.

In terms of the use of CNA as a therapy for VVS, 49% (79/161) of the respondents stated that they did not perform this procedure in the year leading up to the survey. Around one-quarter (23%, 37/161) had conducted between one and five CNAs during that period, while 10% (16/161) had performed from 6 to 10 procedures, and another 10% (16/161) had carried out between 11 and 20 procedures. Finally, 8% (13/161) of the participants reported conducting more than 20 CNA procedures in the past year. Compared with physicians who do not perform CNA, those who have performed CNA procedures during the last year more frequently work in high-volume EP centres with onsite heart surveys available, and they also more frequently perform tilt tests as part of the routine evaluation of VVS (see Supplementary material online, *Table S1*). When questioned about the year they initiated CNA procedures at their centre, the majority of respondents began in 2022 and 2021, as indicated in *Figure 2*.

**Figure 3:**
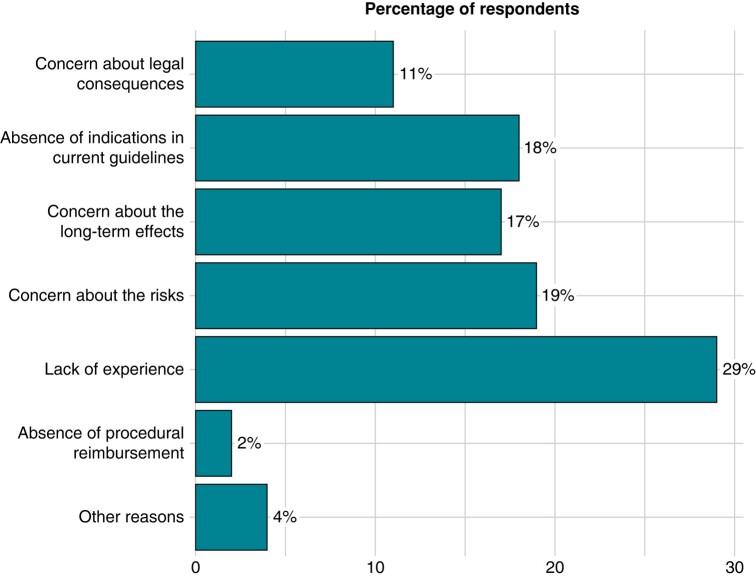
Reasons against CNA. The bar charts display the main reason for not performing CNA in the responding centres as a percentage.

*Figure 3* illustrates the main reservations of those respondents who have not yet adopted CNA as a therapy for VVS. The most frequent reservation was lack of experience, followed by concerns about risk/benefit profile and the lack of coverage by the current guidelines.

Asked about the role of CNA in the next 5–10 years, the majority of the responders (50%, 79/159) imagine a near future in which CNA will be indicated as first-line therapy for patients with recurrent cardioinhibitory VVS; 18% (29/159) of responders believe that CNA will co-exist with pacing for VVS, but not as first-line therapy, whereas 14% (22/159) forecast that it will be a therapeutic option restricted for young patients with recurrent cardioinhibitory VVSs.

### Patients’ selection

Participants to the second part of the survey (reserved for centres currently performing CNA) were asked to identify the most important factors for selecting candidates for CNA in patients with recurrent VVS, the majority (77%, 99/129) emphasized the documentation of spontaneous asystole of vagal origin, including AV block or sinus node arrest. Sixty-four (82/129) of respondents considered the tilt table test as a criterion for patient selection, while patient age was a criterion for 61% (79/129) of participants. Additionally, the response to the atropine test was taken into account by 52% (67/129) of the respondents.

**Figure 4:**
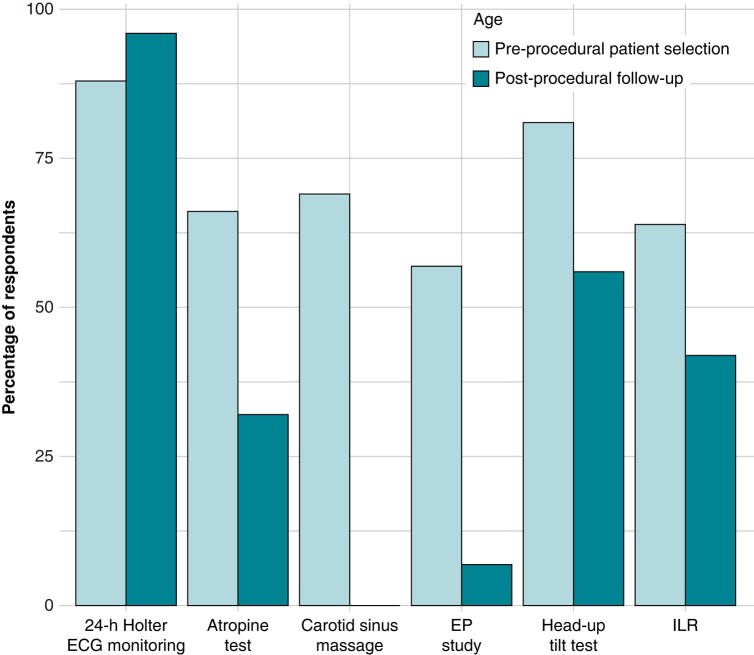
Tests used in the selection and follow-up of patients undergoing CNA. The bar plot displays the percentage of respondents that use 24-h Holter electrocardiogram monitoring, atropine test, carotid sinus massage, EP study, head-up tilt test, or implantable loop recorder for pre-procedural patient selection or patient follow-up. EP study, electrophysiological study; ECG, electrocardiogram; ILR, implantable loop recorder.

**Figure 5:**
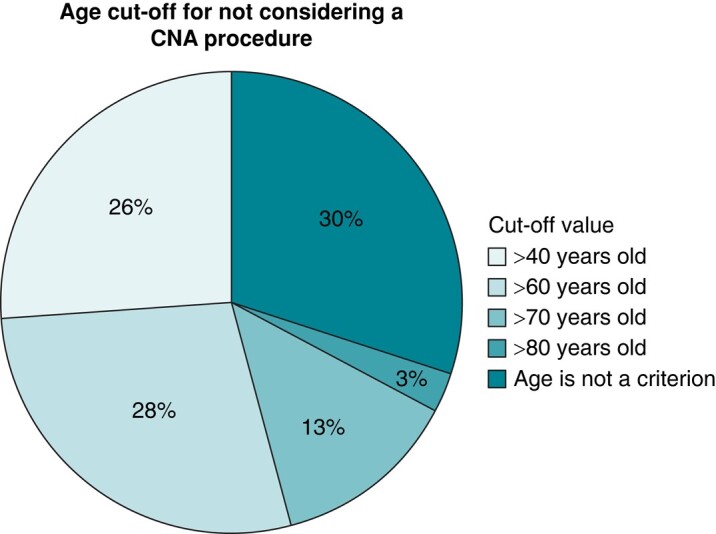
Age limit for not performing a CNA procedure. The pie chart displays the percentage of respondents according to the considered age limit for not performing a CNA procedure. CNA, cardioneuroablation.

*Figure 4* displays the pre-procedural tests typically conducted by the respondents currently performing CNA, 24-h Holter monitoring the most commonly performed pre-procedural assessment, while the invasive EP study is the least frequent. When asked about the significance of patient age in the selection of candidates for CNA, 30% (41/136) of the respondents do not view age as an exclusion criterion for this therapy. Meanwhile, 28% (38/136) consider 60 years old to be the age cut-off point for recommending this therapy, and 26% (35/136) consider 40 years old to be the maximum age for being a suitable candidate for this approach (see *Figure 5*).

### Procedural setting

**Figure 6:**
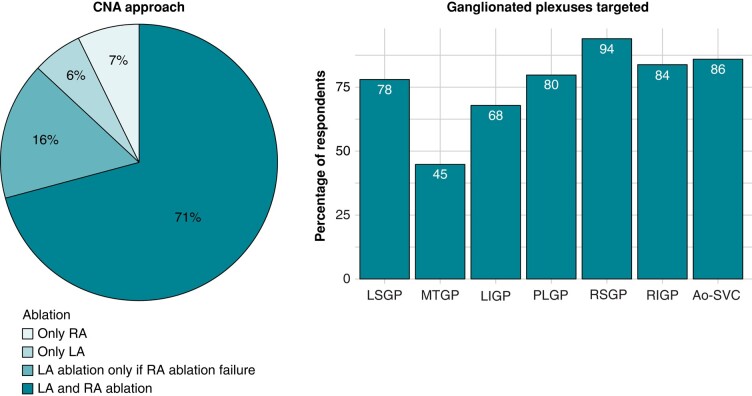
Ablation approach and GPs targeted. The pie chart displays the percentage of respondents according to their ablation approach; the bar chart reported the percentage of respondents according to the GPs targeted during the ablation. Ao-SVC, aorto-superior vena cava; CNA, cardioneuroablation; LA, left atrium; LIGP, left inferior ganglionated plexus; LSGP, left superior ganglionated plexus; MTGP, Marshall tract ganglionated plexus; PLGP, posterior left ganglionated plexus; RA, right atrium; RIGP, right inferior ganglionated plexus; RSGP, right superior ganglionated plexus.

Regarding the procedural setup, 40% (37/93) of physicians perform CNA under general anaesthesia, 34% (32/93) under conscious sedation, and 26% (24/93) under deep sedation. The majority of respondents (71%, 68/96) adopt an approach for targeting GPs in both the right and left atrium as their first-line approach (*Figure 6*). The second most common approach (16%, 15/96) involves performing ablation in the left atrium only in those patients where there is no response following right atrium ablation. Ablation is limited to the right atrium for 7% (7/93) of responders, whereas 6% (6/93) limited the ablation to the left atrium.

*Figure 6* illustrates the GPs that are typically targeted during CNA procedures. The right superior GP is the most commonly targeted, while the Marshall tract GP is the least frequently targeted.

**Figure 7:**
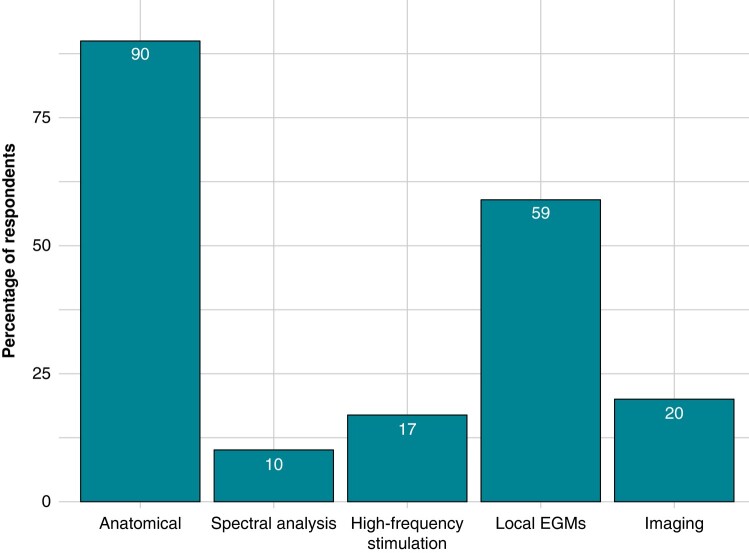
Ganglionated plexuses localization approach. The bar chart displays the percentage of respondents according to the GPs localization approach.

In terms of localizing GPs during the procedure, 90% (83/92) of respondents rely on an anatomical approach, while 59% (54/92) guide the procedure using electrogram (EGM) analysis. Other approaches for GP identification, such as pre-procedural imaging (20%, 18/92), high-frequency stimulation (17%, 16/92), and spectral analysis (10%, 9/92), are less commonly employed by the respondents (see *Figure 7*).

Most of the responders (80%, 74/92) use an ablation catheter with contact force capabilities whereas other ablation technologies like microelectrode catheters are less frequently used (34%, 24/70). The most frequently used power setting ranges between 30 and 40 W (60%, 58/96).

### Procedural endpoints

Participants were asked to indicate the criteria they use to determine that a sufficient amount of radiofrequency (RF) energy has been delivered to a GP. The most common response was to achieve any degree of increase of the heart rate, followed by the elimination of vagal response during the application and by modifying the EGMs. Participants were also asked to indicate the procedural endpoints they used. The most frequent response was the increase in the heart rate, followed by the elimination of the vagal response and finally the lack of response to the atropine test at the end of the procedure.

**Figure 8:**
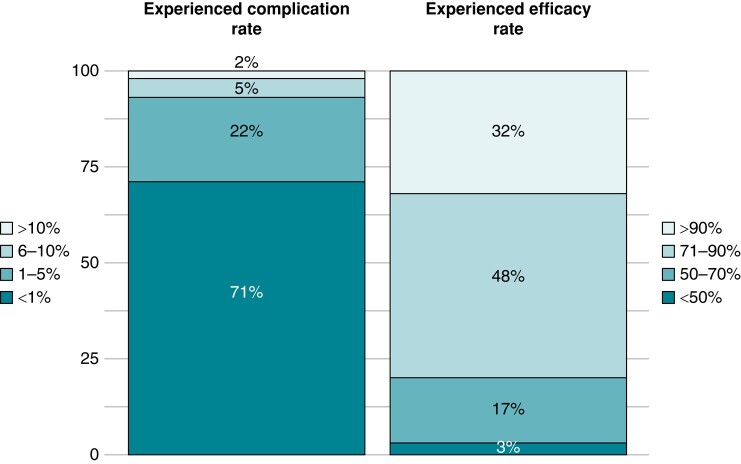
Experienced complication and efficacy rates. The stacked bar chart displays the percentage of respondents according to their experienced complication and efficacy rates.

In terms of safety, the general perception is that CNA is a safe procedure (see *Figure 8*). The estimated rate of procedure-related complications, based on the participants’ experience, is reported as 1% by 71% of respondents and between 1% and 5% for 22%. The most frequent procedure-related complication reported by the respondents was symptomatic sinus tachycardia, followed by vascular complications. None of the respondents reported procedure-related death.

The precautions taken to increase procedural safety were to perform phrenic nerve pacing at high output to monitor phrenic nerve function and avoid phrenic nerve damage (80%, 70/87), to perform a sinus rhythm activation map to avoid RF delivery in the proximity of the sinus node (65%, 56/87), and to place a catheter into the right ventricular apex to avoid symptomatic bradycardia (44%, 38/87).

### Follow-up

Only 11% (11/96) of physicians discharge patients who underwent CNA without anticoagulation or antiplatelet therapy. For the majority of responders (58%, 56/96), patients are discharged with anticoagulation, while 28% (27/96) are discharged on single antiplatelet therapy. Respondents were also questioned about the duration of continuing anticoagulation/antiplatelet therapy after the procedure. Most respondents (55%, 48/88) opt for a 1-month continuation of the therapy after CNA, while 35% (31/88) extend it for at least 2 months.

Regarding the follow-up of patients who underwent CNA, overwhelming majority of respondents routinely perform at least one 24-h Holter monitoring, whereas the EP study is rarely performed, as *Figure 4* shows.

Overall, CNA is regarded as an effective therapy as *Figure 8* shows. About one-third of the respondents (32%, 28/88) estimated the response rate to CNA as exceeding 90%, while 48% (42/88) placed this rate in the range of 71–90%. Only 20% (17/88) of respondents believed the response rate to be below 50%.

The final question of the survey delved into physicians’ approaches to VVS recurrence. Among the responders to this question, 18% (16/89) suggested a redo procedure after the first VVS recurrence, while 45% (40/89) recommended it only after multiple recurrences. One-fifth (18%, 16/89) of participants suggested a redo procedure 3 months after the initial treatment, deeming this period a ‘blanking period’. Lastly, 19% (17/89) of responders opted not to propose a second CNA procedure in the event of VVS recurrence, considering the patient a non-responder to the therapy.

## Discussion

This survey provides valuable insights of the current clinical utilization of CNA in Europe.

### Cardioneuroablation for vasovagal syncope management

The exact role of CNA in the treatment of VVS is still to be determined, as the amount of high-quality evidence-based experimentation remains limited to observational studies and a small randomized trial.^18^ Nevertheless, the results of this survey reflect an enthusiasm for targeted CA as a treatment for recurrent VVS.

Three key observations emerge: (i) Half of the respondents have performed at least one CNA procedure in the last year in their centres. This represents a significantly higher proportion compared to a prior international survey, which reported that only 27% of respondents had personally performed CNA procedures.^10^ (ii) The primary hindrance to initiate a CNA programme is a lack of experience rather than scepticism about its efficacy. (iii) A majority of respondents anticipate that CNA will become the first-line therapy for VVS in the near future.

### Patients’ selection

The majority of existing literature on CNA primarily focuses on young patients,^19,20^ even if recent limited evidence suggests that CNA may be a viable therapeutic option for older patients.^12^ In the present survey, 30% of responders did not consider age as an exclusion criterion for CNA whereas a quarter of the responders consider CNA only suitable for patients under 40 years old. Regarding the indications for CNA, the present survey supports the role of a head-up tilting test for patient selection, in line with the results of a previously published physician-based survey.^10^ Of note, despite the fact that only brief/case reports suggest a benefit of CNA in patients with vagal AV block,^21^ responders considered CNA as a beneficial therapy also in this clinical setting. Conversely, the expected benefit of CNA decreases in case of CSS. This could be attributed to the scarcity of data in the literature.

### Procedural setting

Up to now, there have been no direct head-to-head comparisons between right-sided, left-sided, and bilateral approaches in the context of CA for VVS. A simplified approach focusing solely on right atrial ablation has demonstrated positive outcomes in single-centre registries,^17,22^ although a recent meta-analysis reported that a strategy limited to the right atrium is linked to significantly lower freedom from syncope when compared with left atrial ablation alone or a biatrial approach.^19^ The results of the present survey show that an approach by targeting GPs in both the right and left atrium as their first-line approach is the preferred approach.

Various methods for GP localization have been detailed in the literature.^23–28^ Despite these descriptions, the optimal approach remains to be determined due to the scarcity of high-quality, direct comparison data.^16,29^ The present survey sheds light on the daily practice in this regard. While anatomical-based approaches are the most commonly employed, two-thirds of practitioners guide ablation through EGM analysis. High-frequency stimulation, pre-procedural imaging, and spectral analysis see a more limited use.

### Procedural endpoints and follow-up

While extracardiac vagal stimulation has been outlined to assess vagal denervation,^30^ this survey reveals it is not commonly employed as a procedural endpoint. Simpler methods, such as observing the absence of response to atropine^31^ or an increase in heart rate, predominantly serve as the goals in this context.^14^ It is worth highlighting that these methods should still be standardized to prevent the introduction of subjectivity in the acute evaluation of procedure results. Further studies are needed to better define the acute procedural endpoints.

Descriptions of procedure-related complications are limited,^32^ and the true incidence of complications related to CNA is still to be defined. This survey unveils the perception that CNA is generally considered a safe procedure. Interestingly, based on physician experiences, symptomatic sinus tachycardia emerges as the most frequent complication. Practical insights emerged from this survey include the common practice of discharging patients with anticoagulation for a minimum of 1-month post-procedure. Additionally, 24-h Holter monitoring is the most frequently conducted test during follow-up.^33^ Furthermore, the recommendation for a redo procedure is typically reserved for cases with multiple VVS recurrences after the initial intervention.

### Limitations

The nature of the survey may have introduced a selection bias. Physician already interested in and performing CNA may have primarily participated. Therefore, we cannot exclude a self-selection bias based on interest. Additionally, it is important to keep in mind the potential for recall bias when interpreting the survey results concerning procedural complications and responder rates. Furthermore, since this survey is physician based and anonymous, we lack information regarding respondents beyond what is explicitly requested as part of the survey. Therefore, we cannot dismiss the possibility that multiple physicians from the same centre may have responded, potentially introducing bias. Due to the survey design limitations, we are unable to correlate the approach for GPs ablation (right and/or left atrium) with the anticoagulation therapy at discharge. It is plausible that physicians who opt to discharge patients without anticoagulation more frequently perform an ablation approach restricted to the right atrium.

## Conclusions

This survey offers a snapshot of the current implementation of CNA for VVS treatment in Europe. The results affirm high expectations for the future of CNA, but important heterogeneity exists regarding indications, procedural workflow, and endpoints of CNA. Standardizing protocols for CNA indication, procedure methodology, and outcomes evaluation need to be developed and evaluated in the future.

## Supplementary Material

euae106_Supplementary_Data

## Data Availability

The data that support the findings of this study are available from the corresponding author, upon reasonable request.
