# Supplementary material for: Cardioneuroablation for vasovagal syncope: insights on patients’ selection, centre settings, procedural workflow and endpoints—results from an European Heart Rhythm Association survey
Source: Europace. 2024 May 23;26(5):euae106. doi: 10.1093/europace/euae106 (PMC11114473; doi:10.1093/europace/euae106)
Supplement: euae106_Supplementary_Data [file euae106_supplementary_data.zip › Supplemental Methods.docx]

**Supplemental Methods**

**Center and Arrhythmia Service Characteristics**

Which country are you from?

In which hospital type do you work? Public/Private University Y/N

Is heart surgery available onsite? y/n

Does your hospital have a syncope Unit? y/n

Do you perform Tilt-table test as part of the routine evaluation of syncope? y/n

How many EP procedures are performed at your center/ye­­­­ar?

- Less than 150
- 150 to 500
- 500 to 1000
- More than 1000

How many device implant procedures are performed at your center/year?

- Less than 150
- 150 to 500
- 500 to 1000
- More than 1000

**Management of vagal syncope**

What is your first-line approach for patients over 40 years old with recurrent reflex syncope and one of the following: spontaneous asystolic pauses, tilt test-induced asystolic pauses, or cardioinhibitory carotid sinus syndrome?

- Follow-up with educational recommendations
- Conventional VVI pacemaker implantation
- Conventional DDD pacemaker implantation
- Lead-less pacemaker implantation
- Cardioneuroablation

What is your first-line approach for patients under 40 years old with recurrent reflex syncope and one of the following: spontaneous asystolic pauses, tilt test-induced asystolic pauses, or cardioinhibitory carotid sinus syndrome?

- Follow-up with educational recommendations
- Conventional VVI pacemaker implantation
- Conventional DDD pacemaker implantation
- Lead-least pacemaker implantation
- Cardioneuroablation

In which clinical scenario do you think cardioneuroablation can be useful? **(Multiple answers possible):**

- Recurrent reflex syncope with cardioinhibitory response at tilt test in young patients
- Recurrent reflex syncope with cardioinhibitory response at tilt test in all patients
- Recurrent reflex syncope with documented sinus node arrest
- Recurrent reflex syncope with documented high degree AV block
- Cardioinhibitory carotid sinus syndrome
- Symptomatic bradycardia
- None of them

How many cardioneuroablation procedures did you perform in your center last year?

- None
- 1-5
- 6-10
- 11-20
- More than 20

Which year did you perform the first cardioneurablation at your center (only if not ticked none in question above)?

What is the main motivation why you do NOT perform cardioneuroablation in your center? Multiple answers possible? (Only if question above is “none”)

- Lack of experience
- Concern about the evidence in literature regarding risk/benefit
- Concern about the long-term effects of the procedure
- Absence of indications in the current guidelines
- Concern about legal consequences of a not well stablished intervention
- Other: specify

**Future perspectives**

Which will be the role of cardioneuroablation in the next 5-10 years?

- It will be indicated as first-line therapy for patients with recurrent cardioinhibitory vagal syncope.
- It will be indicated as first-line therapy for patients with functional bradycardia.
- It will be a therapeutic option restricted for young patients.
- It will coexist with pacing but not as a first line therapy
- Don’t know

***Just for centers currently performing cardioneuroablation***

**Patient Selection**

Which do you consider are the most relevant aspects for cardioneuroablation candidates’ selection in patients with recurrent reflex syncope? **(Multiple answers possible):**

- Age
- Type of response at tilt-table test
- Documentation of spontaneous asystole of vagal origin (AV block or sinus node arrest)
- Symptomatic bradycardia
- Response to the atropine test
- Patients’ preference
- Other: specify

Which one of the following tests do you perform for patient selection? **(Multiple answers possible):**

- Tilt test y/n
- Atropine test y/n
- Electrophysiological study y/n
- 24 hours Holter monitoring y/n
- Carotid sinus massage
- Long term monitoring (e.g. ILR) y/n

What do you consider to be the age cut-off point for considering cardioneuroablation in patients with cardioinhibitory reflex syncope?

- More than 40 years old.
- More than 60 years old.
- More than 70 years old.
- More than 80 years old.
- I do not consider age to be an exclusion criterion for cardioneuroablation

**Procedural Setting**

I usually perform cardioneuroablation:

- Under general anesthesia
- Under deep sedation
- Under conscious sedation

Which one is your regular approach for cardioneuroablation?

- Ablation limited to the right atrium
- Ablation limited to the left atrium
- Ablation in the right and left atrium systematically
- Ablation in the left atrium only in those cases without response after right atrium ablation.

Which ganglionated plexuses usually do you target for ablation? (**Multiple answers possible):**

- - The Ao-SVC GP y/n
- - The right superior GP (RSGP) y/n
- - The right inferior GP (RIGP) y/n
- - The posterior left GP (PMLGP) y/n
- - The left superior GP y/n
- - The Marshall tract GP (MTGP) y/n
- - The left inferior GP (LIGP) y/n

Which one is your regular approach for localizing ganglionated plexuses? **(Multiple answers possible):**

- Anatomical approach
- Spectral analysis
- High frequency stimulation
- Local electrogram analysis
- Pre-procedural imaging

What type of catheter do you use?

- Contact force catheter y/n

- Contact/non-contact force with microelectrodes y/n

What is your power setting?

- <30 W 

- 30-40 W 

- 40-50 W 

- >50 W 

Do you use ablation index or lesion size index?

- No, I do not use either.
- Target ablation index value (please provide your maximum value):
- Target lesion size index value (please provide your maximum value):

Your lesion assessment consists of **(multiple answers possible):**

- EGM modification
- Increase in the heart rate
- Elimination of the vagal response
- Amount of radiofrequency delivery (e.g. ablation index)
- It depends on the ganglionated plexus location (vagal response elimination or heart rate increase)
- A combination of all them

Your procedural endpoint consists of **(multiple answers possible):**

- Increase of a predefined percentage of heart rate
- Elimination of the vagal response
- Completion of a predefined number of applications regardless of clinical response
- Lack of response after atropine administration
- Extracardiac vagal stimulation response

Which one of the following approaches do you apply to increase procedural safety? **(Multiple answers possible):**

- I generate a sinus rhythm activation map to avoid sinus node damage y/n
- I generate a phrenic nerve pace-map to avoid phrenic nerve damage y/n
- I deliver RF energy only during simultaneous phrenic nerve pacing wherever it is needed?
- I use a back-up catheter in the right ventricle for pacing to avoid severe bradycardia? y/n

What is your postprocedural management?

- Always ICU/IMC for minimum one night y/n

- Regular ward monitor equipped y/n

- Normal ward without monitoring y/n

**Follow-up**

What is your post-ablation anticoagulation or anti-platelet regime?

- I do not use oral anticoagulation or anti-platelet therapy after the procedure.

- Single APT

- DAPT

- OAC

For how long do you continue oral anticoagulation/anti-platelet therapy?

- 1 week
- 1 month
- 2 months
- More than 2 months

What is the complication rate for this therapy in your personal experience?

I have had the following-complication after performing cardioneuroablation **(multiple answers possible)**:

- Vascular access related
- High degree AV block/need for pacemaker
- Sinus bradycardia
- Symptomatic sinus tachycardia
- Cardiac tamponade
- Phrenic nerve paralysis
- Macro-reentrant tachycardia related with previous ablation sites
- Procedure-related death
- Other

if other: please specify

Which one of the following tests do you perform during the follow-up? Multiple answers possible?

- Tilt-table test y/n
- Atropine test y/n
- Electrophysiological study y/n
- 24 hours Holter monitoring y/n
- Long term monitoring (e.g. ILR) y/n

What is the response rate for this therapy in your personal experience?

- More than 90%
- Range from 70%to 90%
- Range from 50% to 70%
- Less than 50%

What is your approach in case of syncope recurrence?

- I propose a redo procedure after the first recurrence
- I propose a redo procedure only after multiple recurrences
- I propose a redo procedure only after a blanking period of 3 months
- I never propose a redo procedure as I consider the patient a non-responder to cardioneuroablation.
