# Supplementary material for: Cardioneuroablation for vasovagal syncope: insights on patients’ selection, centre settings, procedural workflow and endpoints—results from an European Heart Rhythm Association survey
Source: Europace. 2024 May 23;26(5):euae106. doi: 10.1093/europace/euae106 (PMC11114473; doi:10.1093/europace/euae106)
Supplement: euae106_Supplementary_Data [file euae106_supplementary_data.zip › Supplemental Table 1.docx]

**Supplemental Table 1**. This table that compares the available facility, VVS management, and expectations for CNA therapy in the future between physicians who conducted CNA procedures within the last year and those who did not.

|  | **Performing CNA**  **(n=82)** | **Not Performing CNA (n=79)** | **P-value** |
| --- | --- | --- | --- |
| Syncope Unit (yes) | 49(49/82) | 54 (54/79) | 0.2 |
| Heart Survey available onsite | 71 (71/82) | 57 (57/79) | 0.03 |
| Type of Hospital   - University - Public - Private | 43  17  19 | 38  22  22 | 0.6 |
| Routinely performing Tilt Test | 59 (%) | 42 (%) | 0.015 |
| EP procedure/year   - 0-50 - 51-150 - 151-500 - 501-1000 - >1000 | 0  0  39 (48%)  25 (30%)  18 (22%) | 4 (5%)  1 (1%)  47 (59%)  22 (28%)  5 (6%) | 0.01 |
| Pacing procedure/year   - 0-50 - 51-150 - 151-500 - 501-1000 - >1000 | 1 (1%)  7 (9%)  43 (52%)  23 (28%)  8 (10%) | 0  8 (10%)  49 (62%)  16 (20%)  6 (8%) | 0.57 |
| First-line approach for patients over 40 years with cardioinhibitory VVS   - VVI pacemaker implantation - DDD pacemaker implantation - Lead-less pacemaker implantation - Cardioneuroablation - Educational recommendations | 1 (1%)  20 (24%)  3 (4%)  37 (45%)  21 (26%) | 6 (8%)  42 (53%)  4 (5%)  0  27 (34%) | <0.001 |
| First-line approach for patients under 40 years with cardioinhibitory VVS   - VVI pacemaker implantation - DDD pacemaker implantation - Lead-less pacemaker implantation - Cardioneuroablation - Educational recommendations | 0  3 (4%)  0  57 (70%)  22 (26%) | 2 (3%)  5 (6%)  4 (5%)  6 (8%)  62 (78%) | <0.001 |
| Role of CNA in the next 5-10 years   - First-line for functional bradycardia - Restricted to young patients - Coexist but not as first-line therapy - First-line in cardioinhibitory VVS - I don’t Know | 8 (10%)  9 (11%)  10 (12%)  49 (60%)  6 (7%) | 3 (4%)  13 (16%)  19 (24%)  29 (37%)  15 (19%) | <0.01 |
