# Supplementary figures and images for: Cardioneuroablation for vasovagal syncope: insights on patients’ selection, centre settings, procedural workflow and endpoints—results from an European Heart Rhythm Association survey
Source: Europace. 2024 May 23;26(5):euae106. doi: 10.1093/europace/euae106 (PMC11114473; doi:10.1093/europace/euae106)

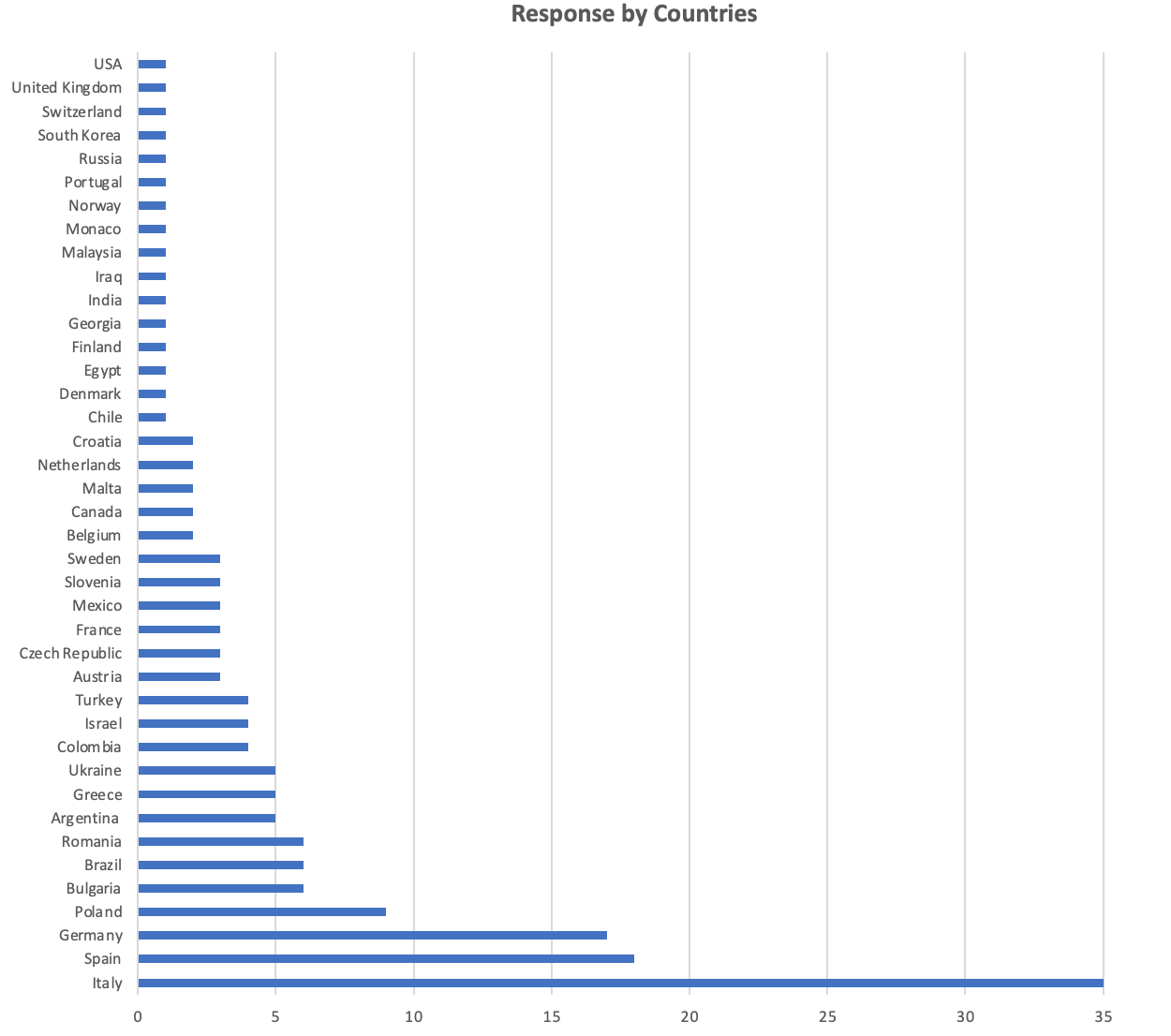

Supplement: euae106_Supplementary_Data [file euae106_supplementary_data.zip › Supplemental Figure 1.png]
